# Enhancing research methods: The role of systematic and scoping reviews in orthopaedics, sports medicine and rehabilitation

**DOI:** 10.1002/jeo2.70069

**Published:** 2024-11-04

**Authors:** Robert Prill, Aleksandra Królikowska, M. Enes Kayaalp, Nikolai Ramadanov, Jon Karlsson, Michael T. Hirschmann

**Affiliations:** ^1^ Center of Orthopaedics and Traumatology University Hospital Brandenburg/Havel, Brandenburg Medical School Theodor Fontane Brandenburg a.d.H. Germany; ^2^ Faculty of Health Sciences Brandenburg, Brandenburg Medical School Theodor Fontane Brandenburg a.d.H. Germany; ^3^ Ergonomics and Biomedical Monitoring Laboratory, Department of Physiotherapy, Faculty of Health Sciences Wroclaw Medical University Wroclaw Poland; ^4^ Istanbul Kartal Research and Training Hospital Istanbul Turkey; ^5^ Department of Orthopaedics Sahlgrenska University Hospital, Sahlgrenska Academy Gothenburg Sweden; ^6^ Department of Orthopaedic Surgery and Traumatology Kantonsspital Baselland (Bruderholz, Liestal, Laufen) Bruderholz Switzerland

## Abstract

Efforts to improve research standards in orthopaedics, sports medicine, physical therapy, and rehabilitation are ongoing. Rrehabilitation andomized Controlled Trials (RCTs) are widely regarded as the gold standard for the collection of primary data. Similarly, systematic reviews, due to their methodological rigor, have become the gold standard for evidence synthesis in medicine. However, while narrative reviews often lack scientific rigor and systematic reviews are not suited to addressing all research questions, other robust review formats are necessary. When the outcomes of interest are unknown or difficult to define in advance, scoping or mapping reviews are more appropriate, as they can address a broader range of questions within the field. Consequently, it is essential to establish rigorous methods for conducting scoping reviews across medical disciplines.

**Abbreviation**
ACL: anterior cruciate ligament

The orthopaedic community puts enormous effort into improving research methods due to a continuously increasing need for rigorous research standards [19]. This effort includes general fields like the external validity of studies, which should be optimized through the adherence of authors to checklists and must be assessed with rigorous risk of bias tools when evaluating a study's quality in the peer review process or when including studies to combine further evidence summaries [20]. Increased validity and reliability of the used measures are also essential to make the measures more trustworthy [9]. Responsiveness over time, when patient‐reported outcome measures are being used, is also essential. The meaningfulness of a study can be enhanced when the results are comparable to other studies through the use of similar measures and if the measures are valid for the issues of interest [1, 2]. While significance is relevant for the generalization of a difference, the effect size of the findings should be used to express the relevance. The minimal clinically important difference should always be considered when relevance is being assessed [11, 17].

The use of systematic reviews with rigorous methods is highly relevant to the field and for the generalization of research findings [18]. In recent times, this has become increasingly evident. While not all findings can be summarized in systematic reviews, the method for evidence synthesis should be as reliable as possible. Narrative reviews might be an option in few cases, but generally, newer forms of precise methods, such as scoping reviews, have a higher potential to contribute to the research process. However, there is currently limited robust guidance available related to the use of scoping reviews [15].

Scoping review methods are less well‐established than systematic review methods, which sometimes raise questions about their robustness. Nevertheless, scoping reviews can notably contribute to the research process by identifying knowledge gaps.

A recent scoping review identified the missing evidence about non‐operative management in patients with femoroacetabular impingement and concomitant Tönnis Grade 2 or more hip osteoarthritis [3]. It has been shown that high‐level soccer players also have a low rate of return to performance after hip arthroscopy for femoroacetabular impingement syndrome [24]. These findings can be understood as a call to action for researchers focusing on the non‐operative treatment of femoroacetabular impingement and may facilitate the publication of well‐done and relevant studies in the field of rehabilitation. In the rehabilitation discipline, scoping reviews are already used relatively frequently. The safe return to sports of athletes is one of the most challenging tasks for rehabilitation specialists, and some questions, such as the significant influence of psychological factors, might already have been answered with systematic reviews [16, 26].

Implementation of the best available evidence into clinical practice plays a crucial role, and some of those findings are already implemented or will be in the near future [7, 21]. Still, more insights from primary and registry studies are needed to support findings related to prevention and the often winding road of return to sport [5, 25]. Sometimes, more open questions than allowed in systematic reviews need to be asked when considering new aspects. For example, the dynamic knee valgus and the role of the gluteal muscles are of significant interest for rehabilitation after knee injury and surgery, but it is often not clear which questions to ask or which studies to conduct [22].

Scoping reviews, a type of knowledge synthesis, aim to map the key concepts, types of evidence and gaps in research within a particular field or topic. Positioned between systematic and narrative reviews, scoping reviews combine the comprehensive and structured approach of systematic reviews with the broad, exploratory nature of narrative reviews. This unique blend allows for detailed literature mapping without the stringent critical appraisal typically required by systematic reviews and goes beyond the level of narrative reviews. As such, alignment strategies in knee arthroplasty represent a complex interplay of various anatomical and dynamic parameters. Narrative reviews can provide an overview and suggest future research directions in this intricate field [8], while systematic reviews can address specific questions by offering targeted answers [23]. The benefits of narrative reviews also apply to procedures that are otherwise unfamiliar to certain audiences around the globe in orthopaedics and sports medicine [29] or to provide current perspectives on topics such as the implementation of artificial intelligence in orthopaedic research [30].

Recently, a series of systematic reviews on anterior cruciate ligament (ACL) reconstruction evaluated various aspects of graft options and outcomes [4, 10, 12, 13, 14, 27, 28]. Current literature supports quadriceps tendons with or without a bone block as safe and viable options for ACL reconstruction, offering comparable clinical outcomes combined with low complication and revision rates [13]. Another review revealed that five‐stranded hamstring autografts, although having larger diameters, yielded clinical outcomes similar to those of four‐stranded hamstring autografts [27]. Regarding donor site morbidity, hamstring and quadriceps tendon autografts had lower morbidity rates than bone‐patellar tendon‐bone autografts [3]. Studies evaluating autografts with and without ligament augmentation showed no significant differences in patient‐reported outcomes, knee laxity restoration or graft failure rates [12]. Regarding the revision ACL reconstruction, comparisons of quadriceps and hamstring tendon grafts revealed similar outcomes regarding patient‐reported results, knee laxity restoration and failure rates [14]. Also, another systematic review and meta‐analysis demonstrated that using quadriceps tendon autografts shows comparable outcomes to hamstring and bone‐patellar tendon‐bone autografts [4]. Research comparing contralateral and ipsilateral hamstring autografts in primary or revision ACL reconstruction found no significant differences in clinical outcomes [28].

In contrast, the scoping review on platelet‐rich plasma augmentation in ACL reconstruction found the evidence too scattered to draw any firm conclusion about its usefulness. However, it also highlighted potential areas for further exploration, sparking curiosity and interest [6].

Taken together, the primary distinction between scoping and systematic reviews lies in their distinct purposes. Scoping reviews, with their broad scope, aim to map the literature and identify knowledge gaps. In contrast, systematic reviews have a narrow and specific scope, aiming for precise, evidence‐based conclusions. Systematic reviews focus on aggregating and analysing data from similar studies, providing a clear and concise understanding of the research landscape. Consequently, there may be more space for high‐quality, content‐driven scoping reviews to ask better questions.

## CONFLICT OF INTEREST STATEMENT

The authors declare no conflict of interest.

## ETHICS STATEMENT

Not applicable.
